# Temporal stability of fecal cortisol metabolites in mountain-dwelling ungulates

**DOI:** 10.1007/s00114-022-01792-y

**Published:** 2022-03-24

**Authors:** Valerio Donini, Elisa Iacona, Luca Pedrotti, Sabine Macho-Maschler, Rupert Palme, Luca Corlatti

**Affiliations:** 1 Stelvio National Park – ERSAF Lombardia, Via De Simoni 42, 23032 Bormio, Italy; 2 Stelvio National Park – Autonomous Province of Trento, Via Roma 65, 38024 Cogolo di Peio, Italy; 3 Department of Biomedical Sciences, Unit of Physiology, Pathophysiology and Experimental Endocrinology, University of Veterinary Medicine Vienna, Veterinärplatz 1, 1210 Vienna, Austria; 4 Chair of Wildlife Ecology and Management, University of Freiburg, Tennenbacher Straße 4, 79106 Freiburg, Germany

**Keywords:** Chamois, Glucocorticoids, Red deer, Sampling design, Stability, Stress, Ungulates

## Abstract

Fecal cortisol metabolites (FCMs) are widely used to track stress responses in wildlife and captive species. Rules of thumb suggest that samples should be collected as soon as possible after defecation, to avoid decay of FCMs. To date, however, only a few studies investigated the stability of defecated FCMs over time, and most of them were conducted in controlled laboratory conditions. Here, we investigated the stability of FCMs over seven consecutive days, in two mountain-dwelling ungulates, under natural environmental conditions using a semi-experimental approach. Fecal samples from Northern chamois *Rupicapra rupicapra* (*n* = 24) and red deer *Cervus elaphus* (*n* = 22) were collected in summer of 2020 within the Stelvio National Park, Italy, and placed in an open area above 2000 m a.s.l. For the next 7 days, we collected a portion of each sample, and all sub-samples were analyzed with an 11-oxoetiocholanolone enzyme immunoassay. Exposure, temperature, and precipitation were fitted as covariates in non-linear generalized mixed models to assess FCM variation over time, and competing models were selected using AICc. For chamois, the best model included only time as a predictor, while for red deer, it included time, precipitation, and exposure. For both species, FCM values decreased rapidly from the first days after deposition until the fourth day. For red deer, in northern-exposed samples, FCM values decreased slower than in south-exposed ones; furthermore, FCM values increased with increasing precipitation. Our results offer a solid methodological basis to wildlife researchers and practitioners interested in the investigation of the ecological factors affecting stress variation in wildlife and support the recommendation to collect samples as fresh as possible, to avoid misleading inference. Further studies are necessary to evaluate the stability of FCMs when other enzyme immunoassays are used.

## Introduction

Glucocorticoids (GC) (cortisol/corticosterone) are widely used as stress indicators in wildlife (Sheriff et al. 2011). When stressful stimuli induce the release of adrenocorticotropic hormone (ACTH), adrenal cortex increases the synthesis and secretion of GCs (Schatz and Palme 2001); it is important to keep in mind, however, that GCs also have basic metabolic functions (Sheriff et al. 2011). GCs are metabolized by the liver and other organs resulting in metabolites excreted in the feces after a species-specific time delay (Palme et al. 2005; Palme 2019). Several sample materials can be used to measure glucocorticoids or their metabolites in feral species, including plasma or blood samples (Morton et al. 1995), saliva (Majchrzak et al., 2015), hair (Salaberger et al., 2016), urine, and feces (Sheriff et al. 2011). Most studies rely on feces collection, because of the non-invasiveness of the method, which does not imply physical capture and therefore is feedback-free (De Clercq et al. 2014).

Fecal cortisol metabolites (FCMs) may be viewed as proxies of stress responses in relation to environmental stimuli (Dehnhard et al. 2001). As high levels of stress may affect individual fitness (Millspaugh and Washburn 2004), e.g., by decreasing immune response and increasing susceptibility to disease (Dhabhar 2014), FCMs can be used as indicators of animal welfare and can improve our understanding of ecological, evolutionary, conservation, and management processes (Sheriff et al. 2011; Palme 2012). For example, FCMs have been used to evaluate the impact of anthropogenic activities (Carbillet et al. 2020), environmental stressors (Corlatti et al. 2011; Hunninck et al. 2020), social environment (Creel 2001; Hadinger et al. 2015), or reproductive status on stress level (Goymann et al. 2001; Dantzer et al. 2010). Furthermore, FCMs represent pooled fractions of plasma glucocorticoids, because fluctuations due to secretory patterns in feces are attenuated (Harper and Austad 2000; Palme 2019). However, challenges in the use of FCMs reside in implementing correct methods for sample collection and storage (Palme 2019). A variety of factors can influence the stability of FCMs after defecation (Möstl et al. 2005; Touma & Palme 2005), such as precipitation, humidity (Washburn and Millspaugh 2002), and ambient temperature (Thiel et al. 2005). Consequently, several authors suggest collecting and storing fecal samples as soon as possible, i.e., immediately after deposition (Möstl et al. 2002; Lexen et al. 2008; Evans et al. 2013). In the field, however, immediate storage is hardly feasible and the time since defecation is usually difficult to determine, at least when sampling is conducted on an anonymous basis (*cf*. Corlatti 2018). For studies of stress ecology, it is therefore important to know the decay pattern of FCMs over time, to establish a valid sampling and storage model of fecal matter. To avoid mistakes in the evaluation of stress levels, deriving from variations in the concentration of FCMs caused by the influence of time and environmental variables, it is essential to know the species-specific temporal stability of FCMs (Millspaugh and Washburn 2004). However, information on the effects of time and natural environmental factors on the stability of fecal FCMs is poor and the results contradictory (Stevenson et al. 2020). Confounding factors can alter the results of FCM analyses, and they must be considered in the analysis to ensure a correct data interpretation (Palme 2019).

Previous studies reported different patterns of FCM stability in a number of species including gelada baboons *Theropithecus gelada*, arctic foxes *Vulpes lagopus*, for which no significant effects of time on FCM concentration was detected (Beehner and Whitten 2004; Larm et al. 2021); domestic sheep *Ovis aries*, where a decreasing or increasing trend in FCM measurements was found, depending upon the used assay (Lexen et al. 2008); southern hairy-nosed wombat *Laisorhinus latifrons*, for which an increase in FCM metabolites was found during in the first few hours after defecation, followed by a decrease in the next hours (Descovich et al. 2012). Notably, only a few studies investigated the pattern of FCM stability under natural environmental conditions. For example, in Western lowland gorilla *Gorilla gorilla gorilla* (Shutt et al. 2014), the pattern of FCM levels showed a linear decreasing trend in the first hours after deposition, while in tiger *Panthera tigris* (Parnell et al. 2015), FCM concentrations significantly increased after 48 h after deposition.

In this study, we investigated the temporal pattern of FCM stability in two mountain-dwelling large herbivores, the Northern chamois *Rupicapra rupicapra* and the red deer *Cervus elaphus* under natural environmental conditions. To date, there are no studies investigating the stability pattern of FCM in ungulates in natural environment and only a few studies under simulated environmental conditions (Washburn & Millspaugh 2002, for white tailed deer *Odocoileus virginianus*; Hadinger et al. 2015 for chamois). In white tailed deer, simulated rain significantly increased FCM values in a time-period of 7 days, while different storage temperature did not impact on FCM stability in the same time-period (Washburn and Millspaugh 2002). In chamois, feces stored at room temperature (daily mean temperature: 6–8 °C), for up to 24 h, showed no general trend in FCM levels (Hadinger et al. 2015).

Based on the existing literature, we hypothesize two possible patterns of stability: (1) drastic reduction in FCM concentration soon after deposition and (2) stable FCM concentration at constant levels in the first few days after deposition.

## Materials and methods

### Study area and populations

**Fig. 1:**
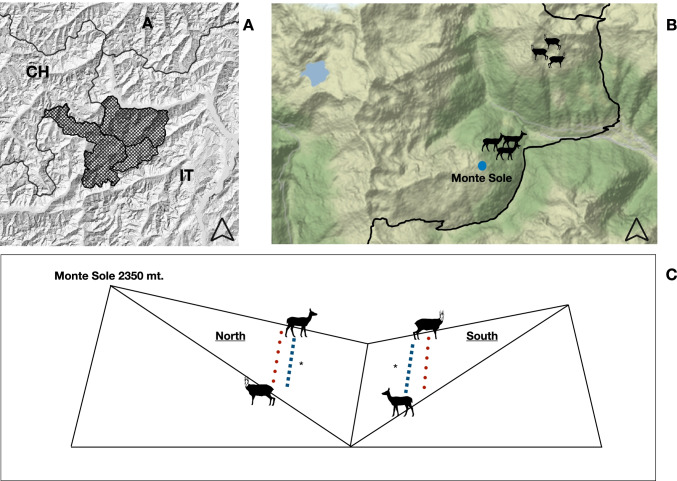
Panel **A** shows the location of the Stelvio National Park. Panel **B** shows the sampling area, within the Trentino sector in the south-eastern part of the Stelvio National Park. Panel **C** shows the location (northern and southern exposure) of the samples deployed to experimentally investigate the temporal stability of fecal cortisol metabolite levels in chamois and red deer. Red dots represent chamois samples, blue dots represent red deer samples; dots are not representative of true number of samples, but only of their approximate location. The asterisk (*) represents the relative location of the temperature-recording devices

The study was conducted in the Trentino sector of the Stelvio National Park, Central Italian Alps (Fig. 1A and B). Climate is alpine, and altitudes range from 1500 to c. 3700 m a.s.l. Below the treeline (< 2100 m a.s.l.), forests are mainly composed of spruce *Picea abies*, larch *Larix decidua*, and Swiss pine *Pinus cembra*; above the treeline, alpine and subalpine meadows are mainly composed by alpine sedge *Carex curvula*, Haller’s fescue *Festuca halleri*, and colored fescue *Festuca varia*. Red deer is one of the most representative species within the Park. After near-extinction in the first half of the nineteenth century, red deer naturally recolonized this area, increasing dramatically in numbers in the recent years, reaching peaks of some 2000 individuals in 2008 (Bonardi et al. 2017) and about 1000 individuals in 2020 (Corlatti et al. 2019). Chamois populations, on the other hand, have been declining in recent years from *c*. 2000 individuals in the 1990s to *c*. 700 individuals in 2019 (Corlatti et al. 2019).

### Data collection and experimental design

During summer of 2020, 24 and 22 feces were collected, respectively, from chamois and red deer. To ensure sampling of fresh feces, samples were collected over two consecutive days, one for each species. At sunrise, we started looking for animals with optical instruments and, when spotted, we waited until defecation and collected the sample from different individuals. Moreover, to avoid sex-biased sampling (Touma and Palme 2005), samples were collected only from females. Immediately after collection, each sample was mixed to ensure homogeneity in FCM concentration within individual scats (Millspaugh and Washburn 2004; Möstl et al. 2005).

Within 2 h from collection, all samples were placed in an open area within the study site, on the top of Monte Sole (2350 m a.s.l.; 10.76477 E, 46.39951 N; Fig. 1C). The samples were exposed to weather conditions without any kind of protection. To assess over-time variation in FCM levels, the following environmental variables were collected. First, for each species, half of the samples were located on a southern exposed slope, and the second half on a northern exposed slope. For the two slopes, a temperature device (iButton Maxim DS1922L) was used to collect information on mean daily temperature (in °C). Precipitation (in millimeter) was calculated, for both slopes, as the cumulative amount of rain from the first day of deployment, retrieved from a meteorological station within the study area (available from www.meteotrentino.it). Starting from the day of deployment, for the 7 consecutive days, a portion of each sample was collected with a spoon, bagged, labeled, and stored frozen at − 20 °C. A total of 230 sub-samples were collected (*n* = 128 for red deer and *n* = 102 for chamois). The number of sub-samples was not consistent across days (for red deer and chamois respectively: day 0, 22 and 24; day 1, 21 and 23; day 2, 21 and 21; day 3, 19 and 17; day 4, 19 and 10; day 5, 15 and 5; day 6, 11 and 2), as for some samples not enough material was left, because feces were eaten by beetles.

### Laboratory analysis

FCM analyses were conducted using an 11-oxoetiocholanolone-17-CMO:BSA enzyme immunoassay (EIA, measures group of FCM with a 5 $\beta$-3 $\alpha$-ol-11-one structure; Möstl et al. 2002), previously validated for red deer and chamois (Anderwald et al. 2021; Huber et al. 2003). These analyses require samples to be fresh and wet. As our samples were collected over 7 days, their freshness and humidity were different among the samples. This required that all samples be dried until constant weight was reached, to ensure homogeneity in the analyses. To assess the dryness of the samples (measured as weight loss by evaporated water), at intervals of 1, 2, 3, 3.5, 4.5, and 16 h, 10 samples of red deer and 10 samples for chamois were weighted. At the end of the 16th hour, weight variation was close to zero. An amount of 0.3 g was taken from each dried sample and mixed with 0.2 ml of water and 5 ml of 80% methanol. Next, samples were vortexed for 30 min with a multi-vortex and then centrifuged for 15 m at 2500 g (Palme et al. 2013). Finally, after the supernatant was transferred, FCMs were analyzed with a group-specific EIA (for details see Möstl et al. 2002).

### Statistical analysis

Regression models were used to investigate the variation of FCM values over time. FCM, as a response variable, is often log-transformed to meet the assumptions of linear models (*cf*. Corlatti 2018). However, generalized models with a gamma conditional distribution and a log-link offer a more natural approach to modeling data that are continuous and strictly positive, such as FCMs. We thus started fitting a “beyond optimal” gamma model (one for each species) where the predictor included the non-linear effect of time (days from scat deposition) in interaction with exposure as explanatory variables, and air temperature and precipitation as control variables. To account for repeated sampling of the same individuals, scat ID was fitted as a random intercept, and each individual line was allowed to have a different slope with respect to day of sampling. The beyond optimal, generalized non-linear-mixed model was thus of the general form:

$${\mathrm{FCM}}_{ij} \sim \mathrm{ Gamma}({\mu }_{ij},\tau )$$

$$\mathrm{E}\left({\mathrm{FCM}}_{ij}\right)= {\mu }_{ij}\mathrm{ and var}({\mathrm{FCM}}_{ij}) = {\mu }_{ij}\times \varphi$$

$$\mathrm{log}({\mu }_{ij}) = {\beta }_{1}+ {\beta }_{2}\times ns\left({\mathrm{day}}_{ij},k\right)+ {\beta }_{3}\times {\mathrm{exposure}}_{ij}+ {\beta }_{4}\times ns\left({\mathrm{day}}_{ij},k\right) :{\mathrm{exposure}}_{ij}+ {\beta }_{5}\times {\mathrm{temperature}}_{ij}+ {\beta }_{6}\times {\mathrm{precipitation}}_{ij}+ {a}_{i}+{b}_{i}\times {\mathrm{day}}_{ij}$$

$${a}_{i} \sim N(0, {\sigma }_{1}^{2})$$

$${b}_{i} \sim N(0, {\sigma }_{1}^{2})$$

where ${\mathrm{FCM}}_{ij}$ was the *j*^th^ observation of individual *i*, which is assumed to follow a gamma distribution with mean ${\mu }_{ij}$ and variance ${\mu }_{ij}\times \varphi$. The term *ns* represent the natural spline with *k* degrees of freedom. Natural splines were used to account for the expected non-linearity of FCM variation over time; this approach was preferred over additive modeling because we did not expect complex non-linear patterns, and natural splines were chosen because they are less erratic than other splines at the boundaries of the data (Perperoglou et al. 2019). Due to paucity of data on the last day of sampling, the dataset was truncated to the 5th day of data collection for chamois, while for red deer, the full dataset was used. For natural splines, *k* was set to 4 in the chamois model and 5 in the red deer model. The term *a*_*i*_ is the random intercept for scat ID, which is assumed to be normally distributed. The term *b*_*i*_ is a random slope, which allows for random variation of scat ID around the slope for sampling day, and is also normally distributed. Prior to fitting the beyond optimal model, we checked for multicollinearity among variables in the predictor through the variance inflation factor (VIF): VIF values < 3 can be generally considered inconsequential (Zuur et al. 2010) and VIF = 5 is often used as a threshold value (Ieno and Zuur 2015). For both species, moderate collinearity among sampling day, exposure, temperature, and precipitation was detected (VIF values respectively 11.5, 5.9, 10.9, and 5.3 for chamois; 6.6, 7.6, 11.6, and 5.0 for red deer); in order to keep proxies of temperature and precipitation in the beyond optimal model for subsequent model selection, both variables were dichotomized into values “lower” and “higher” than their mean, which allowed to mitigate multicollinearity (VIF values for sampling day, exposure, dichotomized temperature, and precipitation were respectively reduced to 3.2, 1.6, 3.1, and 2.2 for chamois; 4.2, 3.0, 3.3, and 4.0 for red deer). The beyond optimal model was fitted by restricted maximum likelihood (REML) and inspected for violation of assumptions through quantile residual plot (Dunn and Smyth 2018).

Given the complexity of the beyond optimal model, we searched for a more parsimonious model using a 3-step model selection approach. First, we found the optimal random structure by comparing the beyond optimal model with a simpler model with random-intercept only, also fitted by REML, by means of a likelihood ratio test (Zuur et al. 2009). Next, we started from the model selected in step 1 to find the optimal non-linear pattern of sampling day, by comparing models with different values of *k* (from the highest value to 1) while keeping all other variables fixed, using the Akaike information criterion corrected for small samples (AICc: Hurvich and Tsai 1989); in step 2, all model were fitted by maximum likelihood (ML). Finally, starting from the model selected in step 2, we performed an automated model selection comparing all combinations of fixed effect terms using the AICc: we provisionally selected models with ΔAICc value < 6; to avoid retention of overly complex models, we then removed models from the candidate set if they were more complex versions of models with a lower AICc (Richards et al. 2011; *cf*. also Burnham and Anderson 2002). The models retained in the candidate set were refitted by REML (Zuur et al. 2009) and inspected for quantile residual distribution (Dunn and Smyth 2018).

Finally, for both species, we inspected random effects by plotting the pattern of individual lines with respect to sampling days. The visual inspection of random effects should provide indications as to whether the pattern of FCM variation over time is consistent among individual samples.

All analyses were conducted with R *v*. 4.0.2 (R Core Team 2020) in RStudio *v*. 1.3.1056 (RStudio Team 2020). VIF values were inspected with the “car” package (Fox and Weisberg 2019). Generalized mixed models were fitted with the package “glmmTMB” (Brooks et al. 2017), using the package “splines” (R Core Team 2020) to account for the non-linear effect of sampling day. Automated model selection was performed with the package “MuMIn” (Bartoń 2020). Parameter estimates were inspected with the package “parameters” (Lüdecke et al. 2020a) and the marginal and conditional *R*^2^ values (Nakagawa and Schielzeth 2013) extracted with the package “performance” (Lüdecke et al. 2020b). The adequacy of quantile residual distribution was assessed with the package “DHARMa” (Hartig 2020). Marginal effects were plotted using the package “effects” (Fox 2003) and “ggplot2” (Wickham 2016). Random effects were plotted with the package sjPlot (Lüdecke 2020).

## Results

**Fig. 2:**
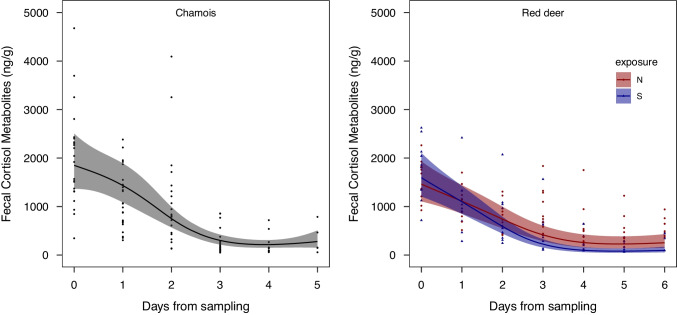
Marginal effects of the model selected to explain the variation in fecal cortisol metabolite levels over time in chamois (left) and red deer (right). Raw data are reported in both panels

For chamois, the optimal random structure was a by-scat ID random intercept with no random slope for sampling day (Table 1: Step 1), while the optimal non-linear pattern of sampling day was a 3rd-degree natural spline (Table 1: Step 2). In the final step, 12 models had delta AICc < 6; 11 of them, however, were more complex versions of the model with the lowest AICc (Table 1: Step 3), thus only the latter was retained for inference. The model included only the significantly negative non-linear effect of sampling day (Table 2) and predicted a drop in levels of FCMs from 1850 ng/g at day 0 to 1434 ng/g at day 1 (− 22%), 751 ng/g at day 2 (− 59%), 302 ng/g at day 3 (− 84%), 216 ng/g at day 4 (− 88%), and 278 ng/g at day 5 (− 85%; Fig. 2). The conditional *R*^2^ for the chamois model was 0.63, while the marginal *R*^2^ was 0.53.

**Table 1:** Model selection procedure used to explain the pattern of decay of fecal cortisol metabolite levels in chamois (on top) and red deer (on bottom) over time. The table reports the different selection steps (see text for details), the fixed and random structure of the models, the estimation method (maximum likelihood [ML] or restricted maximum likelihood [REML]), the selection method (likelihood ratio test [LRT] or Akaike information criterion corrected for small samples [AICc]), and the results of different selection procedures (*P*-value or AICc values). In step 3, only models with delta AICc < 6 are shown. The symbols “*” and “ + ” respectively indicate interactive and additive effects. “ns,” natural spline. Models selected for inference are shown in bold

	Fixed structure	Random structure	Method	Selection	Result
*Chamois*					
Step 1	~ ns(day,4) * exposure + temperature + precipitation	(Day | scat ID)	REML		
	~ ns(day,4) * exposure + temperature + precipitation	(1 | scat ID)	REML	LRT	*P* = 0.318
Step 2	~ ns(day,4) * exposure + temperature + precipitation	(1 | scat ID)	ML	AICc	1543.7
	~ ns(day,3) * exposure + temperature + precipitation	(1 | scat ID)	ML		1542.4
	~ ns(day,2) * exposure + temperature + precipitation	(1 | scat ID)	ML		1551.4
	~ day * exposure + temperature + precipitation	(1 | scat ID)	ML		1548.7
Step 3	** ~ ns(day,3)**	**(1 | scat ID)**	ML	AICc	1539.1
	~ ns(day,3) + exposure	(1 | scat ID)	ML		1539.3
	~ ns(day,3) + temperature	(1 | scat ID)	ML		1539.5
	~ ns(day,3) + exposure + temperature	(1 | scat ID)	ML		1541.0
	~ ns(day,3) + temperature + precipitation	(1 | scat ID)	ML		1541.2
	~ ns(day,3) + precipitation	(1 | scat ID)	ML		1541.2
	~ ns(day,3) + exposure + precipitation	(1 | scat ID)	ML		1541.4
	~ ns(day,3) * exposure	(1 | scat ID)	ML		1542.0
	~ ns(day,3) * exposure + temperature	(1 | scat ID)	ML		1542.3
	~ ns(day,3) * exposure + temperature + precipitation	(1 | scat ID)	ML		1542.4
	~ ns(day,3) + exposure + temperature + precipitation	(1 | scat ID)	ML		1542.8
	~ ns(day,3) * exposure + precipitation	(1 | scat ID)	ML		1543.8
*Red deer*					
Step 1	~ ns(day,5) * exposure + temperature + precipitation	(Day | scat ID)	REML		
	~ ns(day,5) * exposure + temperature + precipitation	(1 | scat ID)	REML	LRT	*P* = 0.012
Step 2	~ ns(day,5) * exposure + temperature + precipitation	(Day | scat ID)	ML	AICc	1782.2
	~ ns(day,4) * exposure + temperature + precipitation	(Day | scat ID)	ML		1780.4
	~ ns(day,3) * exposure + temperature + precipitation	(Day | scat ID)	ML		1780.1
	~ ns(day,2) * exposure + temperature + precipitation	(Day | scat ID)	ML		1795.9
	~ day * exposure + temperature + precipitation	(Day | scat ID)	ML		1797.6
Step 3	~ ns(day,3) * exposure + precipitation	**(Day | scat ID)**	ML	AICc	1773.2
	~ ns(day,3) * exposure + temperature + precipitation	(Day | scat ID)	ML		1774.6

**Table 2:** Parameter estimates of the model selected to explain the pattern of decay of fecal cortisol metabolite levels in chamois (on top) and red deer (on bottom), fitted by restricted maximum likelihood (REML). The table reports the estimates of the regression coefficient and the associated lower (95LCL) and upper (95UCL) levels of the 95% confidence interval

Parameter	Coefficient	95LCL	95UCL
*Chamois*			
Intercept	7.523	7.224	7.822
Day [1^st^ degree]	− 2.391	− 3.030	− 1.752
Day [2^nd^ degree]	− 2.354	− 3.092	− 1.617
Day [3^rd^ degree]	− 1.717	− 2.298	− 1.137
*Red deer*			
Intercept	7.976	7.544	8.408
Day [1^st^ degree]	− 1.894	− 2.486	− 1.303
Day [2^nd^ degree]	− 2.278	− 2.926	− 1.630
Day [3^rd^ degree]	− 1.465	− 1.921	− 1.009
Exposure [South vs. North]	0.087	− 0.304	0.477
Precipitation [low vs. high]	− 0.688	− 1.013	− 0.363
Day [1^st^ degree]: exposure [South vs. North]	− 1.228	− 1.736	− 0.720
Day [2^nd^ degree]: exposure [South vs. North]	− 1.248	− 2.032	− 0.464
Day [3^rd^ degree]: exposure [South vs. North]	− 0.958	− 1.483	− 0.434

For red deer, the optimal random structure was a by-scat ID random intercept with random slope for sampling day (Table 1: Step 1), while the optimal non-linear pattern of sampling day was a 3rd-degree natural spline (Table 1: Step 2). In the final step, only 2 models had delta AICc < 6, but only the top-ranked model was retained for inference as it was nested within the second-best model. The model included the significantly negative and non-linear effect of sampling day in interaction with exposure and the significant effect of precipitation (Table 2). When holding precipitation constant at its low level (average 0.8 mm), the best model predicted a steeper decline in FCM levels for the southern-exposed than for the northern-exposed samples. Specifically, for the southern-exposed samples, FCM predicted values decreased from 1595 ng/g at day 0 to 1099 ng/g at day 1 (− 31%), 591 ng/g at day 2 (− 63%), 224 ng/g at day 3 (− 86%), 98 ng/g at day 4 (− 94%), 80 ng/g at day 5 (− 95%), and 97 ng/g at day 6 (− 94%; Fig. 2). For the northern-exposed samples, FCM predicted values decreased from 1463 ng/g at day 0 to 1110 ng/g at day 1 (− 24%), 741 ng/g at day 2 (− 49%), 416 ng/g at day 3 (− 72%), 258 ng/g at day 4 (− 82%), 228 ng/g at day 5 (− 84%), and 254 ng/g at day 6 (− 83%) (Fig. 2). The conditional *R*^2^ for the red deer model was 0.87, while the marginal *R*^2^ was 0.67.

**Fig. 3:**
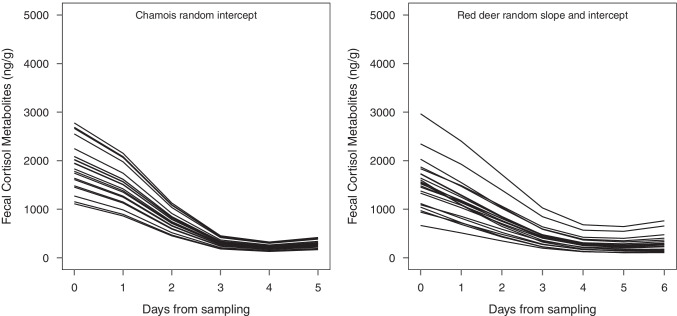
Random effects of the model selected to explain variation in fecal cortisol metabolite levels over time in chamois (left) and red deer (right)

The plots of random effects did not suggest major variations in the individual pattern of decay of FCM values in both species, though in red deer individual heterogeneity in overall level of FMCs was greater than in chamois (Fig. 3).

For both species, the beyond optimal models and the final models did not show issues of convergence, and the quantile residuals were unsystematically distributed, thereby supporting the goodness of the starting and selected models.

## Discussion

Our study investigated the stability of fecal cortisol metabolites over a time period of 7 days in two species of wild ungulates, under natural field conditions. Results show that FCM levels in chamois and red deer steadily decreased over the 7 days of sampling. More specifically, in both species, FCM levels showed a rapid decrease from the first day after deposition to up to the 4th day, and stable levels afterwards. We also found an effect of exposure in red deer, where FCM levels decreased at a slower rate in the northern-exposed site than in the southern-exposed one. Moreover, red deer FCMs increased with increasing levels of precipitation.

To our knowledge, only a few studies have been conducted on the temporal variation of FCM levels in ungulates, and all in laboratories under simulated (controlled) environmental conditions. The controlled experiment on FCMs variation in white-tailed deer, for example, revealed that simulated environmental conditions (different storage temperature) had no effects on FCM levels over 7 days. Similarly, a study conducted on alpine chamois showed stability in FCM levels over a 12-h period (Hadinger et al., 2015). Lab studies offer the advantage of allowing to assess the effect of one variable at a time, while keeping all other variables constant. It is worth noting, however, that FCMs are widely used as indicators of stress responses in a variety of wildlife species under natural conditions (Washburn & Millspaugh, 2004). Therefore, assessing the pattern of stability over time under natural field conditions may provide useful information when defining the sampling design. The results of our field experiment partially contrast with earlier studies, where no apparent pattern of time variation in FCM values was detected, either in ungulates (Washburn & Millspaugh 2002; Hadinger et al. 2015), baboons (*Papio spp.*; Beehner & Whitten 2004), or marsupials (*Macrotis lagotis*; Evans et al. 2013). Similar results, on the other hand, were found in sheep, albeit in a temperature-controlled experiment (Lexen et al., 2008). It is possible that such heterogeneity of results might be partly explained by species-specific variability in FCM stability. Although rules of thumb suggest that fecal samples should be collected as fresh as possible (Washburn & Millspaugh 2002), thus far, this was largely a heuristic recommendation that, at least for ungulates, required empirical support in the field. Based on a semi-experimental approach under natural conditions, we were able to support the recommendation that fecal samples must be collected as soon as possible after defecation, to avoid sampling bias in the assessment of stress variation.

Under natural conditions, many confounding factors can influence FCMs analyses simultaneously (Palme 2019) and, consequently, the results of stability studies (Millspaugh and Washburn 2004; Hadinger et al. 2015). For example, under controlled conditions, a strong increase of FCM levels in feces of white-tailed deer exposed to simulated precipitation was found (Washburn and Millspaugh 2002). Similarly, in a simulated experiment conducted on FCMs in Cheetah *Acinonyx jubatus*, an effect of different drying methodologies of the samples on FCM values over 7 days was found (Terio et al. 2002). Furthermore, a strong effect of individuals and season was found in alpine chamois (Hadinger et al. 2015). Contrary to the time-effect, the effect of precipitation on FCM values in this study was in line with the results of Washburn and Millspaugh (2002), where the daily addition of a constant amount of water increased the levels of FCM in white-tailed deer. The effects of rain on feces can be traced to microbial actions that can alter, metabolize, or degrade steroids (Möstl et al. 1999, Möstl et al. 2005; Lexen et al. 2008), leading to an increase in metabolite concentrations with increasing precipitation (Terio et al. 2002; Washburn and Millspaugh 2002; Millspaugh and Washburn 2004). The differences in FCM levels in relation to exposure site are novel and reveal that the north-facing samples may have been protected from external agents such as sun and heat, which might have reduced FCM variation over time.

Our results should be interpreted with caution. For example, in our analysis, we had some collinearity issues, and consequently, temperature and precipitation had to be dichotomized, thereby reducing their explanatory power. This represents a potential limitation, because it is well known that these environmental variables may simultaneously affect FCM levels (Terio et al. 2002; Washburn and Millspaugh 2002; Millspaugh and Washburn 2004), alongside with other variables such as humidity, wind, or solar radiation. Moreover, after collection, samples had to be mixed to ensure homogeneity within scats, but this change of status (from pellets to homogeneous mixture) may have modified bacterial communities and the potential effect of climatic conditions. Finally, most of the immunoassays used are more or less “group specific” and detect a specific three-dimensional configuration of a part of the steroid (Möstl et al. 2005; Palme 2019). Therefore, using different assays may lead to different results with respect to FCM concentrations. For example, if the assay is specific for the 11-oxo configuration, and microorganisms convert the 11-oxo position into a 11ß-hydroxy configuration, the 11-oxoetiocholanolone assay will not detect this metabolite. Consequently, an assay for the 11ß-hydroxy configuration may show an increase in these metabolites during storage. In fact, in a study conducted in sheep, different EIAs led to opposite trends (decreasing versus increasing FCMs levels: Lexen et al. 2008). Accordingly, our results require confirmation from other assays and, more generally, further studies are necessary to evaluate the stability of FCMs when other enzyme immunoassays are used. Internal variables, which may influence GC metabolites values, such as individual heterogeneity (Morméde et al. 2007), age and sex (Palme 2019), as well as diet (Wasser et al. 1993) and reproductive status (Palme 2019), on the other hand, should not impact our results, as sampling was conducted on the same sex and age class, outside of the reproductive season, and individual heterogeneity is of no concern, at least in chamois (Corlatti 2018).

The results of our work allowed to unravel temporal patterns of FCM stability in two key wildlife species and offer a solid basis to wildlife researchers and practitioners interested in the investigation of the ecological factors affecting stress variations in wildlife, when planning their sampling design. Our investigation was conducted on a relatively coarse (daily) scale, and future studies should focus on the investigation of stability under natural conditions, but at finer temporal scale, for example on an hourly basis (*cf*. Descovich et al. 2012; Hadinger et al. 2015). For all practical purposes, however, it is recommendable to collect fecal samples as fresh as possible, immediately after deposition.

## Data Availability

Data are available and the codes used for analysis are available from the corresponding author upon acceptance.
